# Efficacy and safety of surfactant administration by MIST and INSURE techniques in Neonates with Respiratory Distress Syndrome: A randomized controlled trial

**DOI:** 10.12669/pjms.39.3.7283

**Published:** 2023

**Authors:** Ammara Kaleem, Farah Haroon, Bushra Fatima, Gideon Victor, Mazhar Qadir, Khawaja Ahmed Irfan Waheed

**Affiliations:** 1 Ammara Kaleem Fellow of Neonatology University of Child Health Sciences, Lahore, Pakistan; 2 Farah Haroon Associate Professor of Neonatology University of Child Health Sciences, Lahore, Pakistan; 3 Bushra Fatima Assistant Professor of Neonatology University of Child Health Sciences, Lahore, Pakistan; 4 Gideon Victor Assistant Professor of Neonatology, Shifa Tameer-e-Millat University, Islamabad, Pakistan; 5 Mazhar Qadir University of Child Health Sciences, Lahore, Pakistan; 6 Khawaja Ahmed Irfan Waheed Professor of Neonatology University of Child Health Sciences, Lahore, Pakistan

**Keywords:** MIST, INSURE, Neonates, Respiratory distress syndrome, RCT, Surfactant therapy, Preterm

## Abstract

**Objective:**

To measure the efficacy and safety of surfactant administered by MIST and INSURE to neonates with respiratory distress syndrome.

**Methods:**

A randomized controlled trial was conducted from June 2021 to August 2022 at the NICU of the University of Child Health Sciences, Lahore. Neonates meeting inclusion criteria i.e with RDS who worsened on nasal Continuous positive airway pressure (nCPAP) (fiO2 30%, pressure 6cmH2O) were enrolled in the study in both interventional arms (MIST, n=36 and INSURE, n=36) using simple random sampling. Data was analysed using SPSS 25.

**Results:**

The mean age of neonates in MIST was 1.27±0.40 days and 1.23±0.48 days in INSURE cohort. Neonates with MIST (n=8) required statistically significant reduced need for IMV than INSURE (n=17) technique (P-Value 0.047). This study could not achieve significant difference in duration of mechanical ventilation (1±1.67; 1.52±1.40 days, P=0.152) and duration of nCPAP (3.27±1.65;3.67±1.64 hrs, P=0.312) in MIST versus INSURE. The second dose of surfactant was administered in fewer cases in MIST (n=2) than INSURE (n=7) (P=0.075). Risk estimation, although not significant, determined less likelihood for the pulmonary haemorrhage (0.908 than 1.095), intraventricular hemorrhage (0.657 than 1.353), administration of the second dose of surfactant (0.412 than 1.690) and greater likelihood of discharge (1.082 than 0.270) at 95% confidence interval with MIST technique.

**Conclusion:**

Surfactant therapy through MIST is effective and there is significantly reduced need of IMV than in INSURE. Safety profile though could not achieve statistical significance yet determines less risk of complications associated with MIST than INSURE.

***RCT Registration Number:*** TCTR20210627001

## INTRODUCTION

Respiratory distress syndrome (RDS) is caused by surfactant deficiency and it is defined as respiratory distress within six hours of the birth in the presence of an evidence of hypoxia or requirement of supplementary oxygen to maintain PaO_2_ >50mmHg and specific radiographic findings. RDS presents with tachypnoea, intercostal/subcostal recessions, tracheal tug, grunting and cyanosis. Risk factors for RDS include prematurity^1^, maternal diabetes, caesarean delivery and asphyxia. The incidence of RDS in admitted neonates is variable in different countries which include 1.64% in Saudi Arabia^2^, 2.42% in India^3^, and 8.1 % in Ethiopia.^4^

United States reported that 6.4 neonates per 1000 live births had RDS.^5^ In one report from Pakistan incidence of RDS is 1.2% in very low birth weight neonates.^6^ Multiple strategies such as antenatal steroids, surfactant administration, oxygenation and nasal CPAP have improved the outcome of respiratory distress syndrome.^7^,^8^ Surfactant administration alone has lessened mortalities from RDS by 50%.^9^ Currently, different methods of surfactant instillation are in use such as INSURE (INtubation, SURfactant administration and Extubation to CPAP), LISA (Less Invasive Surfactant Administration) and MIST (Minimally Invasive Surfactant Therapy). INSURE is an effective way of surfactant therapy and also avoids the need for prolonged invasive mechanical ventilation (after initial extubation).

However, sedation, local trauma due to endotracheal tube insertion and extubation failure are major hazards linked with INSURE.^10^,^11^ MIST refers to the administration of surfactant without endotracheal intubation and it is performed through semi-rigid catheters (Angiocath, Surfcath), laryngeal mask airways or inhalation devices. While soft catheters are used in LISA that require help of Magill forceps in addition to laryngoscope for insertion of catheter in trachea when compared with INSURE, MIST has fewer complications including the rate of intubation mortality, PDA, and pneumothorax.^12^-^15^

On the other hand, more time is consumed in MIST technique^14^ than INSURE and there are also risks of laryngoscopy associated complications. Previously, some trials favouring MIST had certain limitations for example small sample size and sedation during the procedure. While few other clinical trials prove no obvious advantage of using thin catheters over INSURE.^16^,^17^ INSURE is still being practiced more commonly than MIST for surfactant administration. This study was aimed to compare the efficacy and safety of surfactant administration by MIST and INSURE in a tertiary care hospital.

## METHODS

It was a prospective, randomized controlled trial performed at the NICU of University of Child Health Sciences, the Children’s Hospital Lahore from June 2021 to August 2022. The study was approved by the Institutional Review Board of UCHS (Ref: 2021-293-CHICH, Dated: 11-06-2021) and informed and written consent was obtained from a parent/guardian. Neonates less than 34 weeks of gestation having respiratory distress, that worsened on nCPAP with FIO_2_ >30 % and 6cm H_2_O pressure^7^ were included. We did not include babies who were intubated in the delivery room or had birth asphyxia. We excluded those babies who had early onset sepsis, congenital heart/ lung abnormality and/or neuromuscular disease. Those who developed sepsis or necrotizing enterocolitis later, were not excluded. The babies were randomized in two groups using online software^18^ by a person not involved in the study.

The MIST technique used direct laryngoscope a six Fr semi-rigid catheter (Surfcath) that was inserted 1-2cm beyond the vocal cords and after catheter placement, the laryngoscope was removed. Following complete dose administration in 5-10 minutes, the semi-rigid catheter was also withdrawn while nCPAP was continued as respiratory support. In INSURE technique; neonates were intubated with appropriate size endotracheal tube and following surfactant instillation, positive pressure ventilation was provided. Babies were extubated after achievement of target oxygen saturation and put on nCPAP.

Vital signs monitoring, principles and precautions for surfactant administration were followed for both techniques as per NICU protocol.^19^ No sedation or premedication was used before the procedure and Poractant alpha (Curosurf) 200 mg/kg was administered. If the infant needed FiO2 more than 30% after six hours of initial surfactant therapy, a second dose of surfactant was given by the same technique. The primary outcomes of this trial were to compare the need for invasive ventilation, duration of mechanical ventilation, duration of nCPAP, oxygen demand and outcome i.e., discharge or death.

While secondary outcomes were length of hospital stay, need for a second dose of surfactant and complications including pulmonary and intraventricular haemorrhage. The sample size was calculated using G*Power software based on the effectiveness of MIST reported in the published studies and the 20% potential dropout rate. The effect size i.e., d=0.65, alpha 0.05, power 80% and allocation ratio=1 determined 72 patients (36 in MIST and 36 in INSURE) interventional arms.

### Data management & analysis:

Data was coded and entered in SPSS v25 for analysis. Demographic variables were described using descriptive statistics. Categorical variables were compared using Chi-square and continuous variables with student’s t-test to determine significance and P-value < 0.05 was considered significant. The relative risk of the outcomes between the two arms was calculated, along with 95% confidence intervals.

## RESULTS

Demographic variables of total 72 babies and 36 in each limb were similar and showed no significant difference between the two groups that are given in Table-I.

**Table-I:** Demographic variables

		Technique		P-Value

		MIST (n=36)	INSURE(n=36)	
Gestational Diabetes	Yes	3	4	0.50
	No	33	32	
Twin delivery	Yes	2	4	0.39
	No	34	32	
Mode of Delivery	SVD	23	19	0.23
	LSCS	13	17	
Sex	Female	13	14	0.80
	Male	23	22	
Gestational Age (weeks)	Min-Max	26.00 – 34.00	27.00 – 34.00	0.30
Age	Mean±SD	1.28±0.46	1.28±0.55	0.981
	Min - Max	0.90 – 2.00	1.00 – 3.00	
Weight	Mean±SD	1.37±0.36	1.40±0.38	0.678
	Min - Max	0.80 – 2.20	1.00 – 2.40	

The Table-II represents the primary and secondary outcomes. There need for IMV was significantly lesser in MIST group than in INSURE (P-Value 0.047).

**Table-II (a):** Primary outcomes.

Measurements		Technique		Df	P-Value

		MIST	INSURE		
Need for invasive mechanical ventilation	Yes(n)	8	17	1	0.047*
	No(n)	28	19		
Duration of invasive mechanical ventilation	Mean	1.00	1.52	58	0.152**
	SD	1.67	1.4		
Duration of nCPAP	Mean	3.27	3.67	68	0.312**
	SD	1.65	1.64		
Outcome	Discharge(n)	28	27	2	0.781*
	Death(n)	8	9		

Table-II (b): Secondary outcomes					

Length of hospitalization	Mean	9.30	9.80	58	0.604**
	SD	4.14	3.51		
Pulmonary hemorrhage	Yes(n)	6	7	1	0.759*
	No(n)	30	29		
Intraventricular haemorrhage	Yes(n)	1	2	1	0.555*
	No(n)	35	34		
Second surfactant dose	Yes(n)	2	7	1	0.075*
	No(n)	34	29		

* Chi-square;

** independent t-test; n=number.

Though not significant the risk estimation analysis determined less likelihood for the need for mechanical ventilation, pulmonary hemorrhage, intraventricular hemorrhage, administration of second dose of surfactant and greater likelihood of discharge with MIST technique (Table-III).

**Table-III:** Risk Estimation of MIST and INSURE by Odds Ratio.

Measurement	MIST		INSURE	

	Value	95% CI Lower-Upper	Value	95% CI Lower-Upper
Need for mechanical ventilation	0.590	0.330-1.06	1.583	1.016-2.470
Pulmonary haemorrhage	0.908	0.479-1.171	1.095	0.622-1.930
Intraventricular haemorrhage	0.657	0.130-3.310	1.353	0.587-3.120
Second surfactant dose	0.412	0.119-1.430	1.690	1.088-2.620
Outcome [discharge – death]	1.082	0.614-1.910	0.270	0.550-1.560

*CI (Confidence interval)

The Fig.2 shows that Neonates in the MIST group experienced lesser oxygen demand (FiO_2_) after one hour of procedure than in INSURE technique (P-value 0.007).

**Fig.1:**
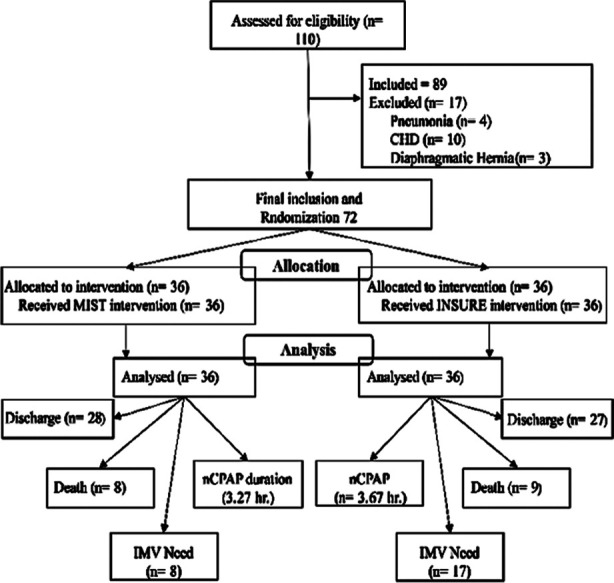
Recruitment and selection of study participants.

**Fig.2:**
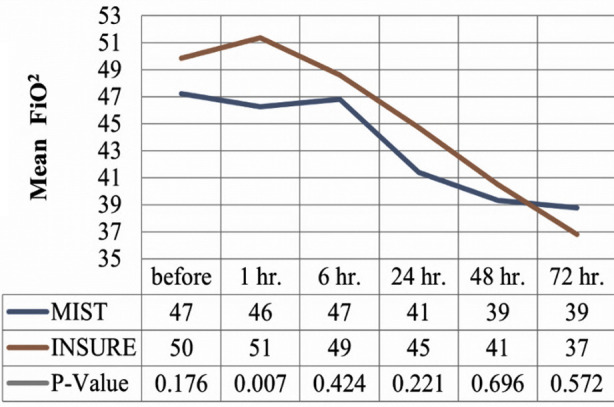
Effect of MIST and INSURE on FiO_2_ level.

## DISCUSSION

Different techniques for surfactant administration have been evaluated in many clinical trials. We compared safety and efficacy of MIST and INSURE in <34 weeks neonate. The age of neonates described in many previous studies^9^,^15^,^16^ was usually within six hours whereas we observed higher mean age in both groups which is mainly because that this trial was performed in a referral center where about 50% of patients presented after six hours of life.

Dargaville et al.^12^, Wang et al.^13^ and others^20^,^21^ established statistically significant lesser need for IMV in MIST group as compared to INSURE which is also evident in our study. However, Gupta et al. had contradictory results and showed no effect of either technique on need for invasive mechanical ventilation^16^ that was thought of probably due to using NIPPV after surfactant administration.

FIO_2_ patterns studied by Mosayebi et al and Halim A et al showed reduced FiO_2_ requirement with MIST than with INSURE,^22^,^23^ we also found similar results at one hour after surfactant instillation. It was because of less desaturations and apnea observed during the MIST technique that is explainable due to use of nCPAP during the procedure. We experienced 90% success rate for administering surfactant in first attempt.

Bao et al. and Sabzehei et al. could not ground significant difference in duration of nCPAP and IMV in both groups likewise reported in our study.^17^,^22^ On the other hand, majority of the trials^12^,^13^,^20^-^22^ show reduced duration of nCPAP and IMV in MIST group than in INSURE group which might be due to different settings and different type of catheters used for MIST. We observed there was relatively shorter duration of hospital stay with MIST technique in comparison with INSURE though it could not achieve value of significance which is consistent with results shown by Mosayebi et al.^22^

Previously experimental trials conducted by Dargaville et al, Wang et al and Han et al determined low rate of complications with variable incidence, in MIST group than in INSURE^12^-^14^ such as in our study. These results might be related to using nCPAP during surfactant administration while our results are contrary to the studies conducted by Gupta et al and Sabzehei, reporting no significant difference in IVH and pneumothorax.^16^,^24^ Feeding tube was used in both the trials whereas we used semi-rigid catheter. Further Sabzehei used caffeine for all patients and NIPPV was used by Gupta which could produce variability in results.

Pulmonary hemorrhage followed by PDA and IVH were common in our study groups as while pneumothorax and PIE were not detected in this study. Though there was no significant difference in complications of surfactant therapy including IVH¸ Pneumothorax and, PDA in control and intervention group yet less likelihood ratio for these complications were noted in risk assessment, such as pulmonary haemorrhage, intraventricular haemorrhage and administration of second dose of surfactant. In this trial, MIST comparatively showed favorable clinical outcome as evident from lesser need for IMV and greater likelihood of discharge.

### Limitations:

This trial has certain limitations such as late presentation delayed the time for first dose of surfactant administration, in some patients, even on second day of life. Further, outcome was not only depending on the surfactant technique or time of administration but also other factors such as NEC and sepsis modified the results. There is need for further studies on large sample size minimizing bias between the two groups.

## CONCLUSION

Surfactant therapy through MIST is effective and there is significantly reduced need of IMV than in INSURE. Safety profile though could not achieve statistical significance yet determines less risk of complications associated with MIST than INSURE.
